# Complete Genome Sequence of *Bacilli bacterium* Strain VT-13-104 Isolated from the Intestine of a Patient with Duodenal Cancer

**DOI:** 10.1128/genomeA.00705-15

**Published:** 2015-07-02

**Authors:** George Tetz, Victor Tetz

**Affiliations:** Institute of Human Microbiology, LLC, New York, New York, USA

## Abstract

We report the complete genome sequence of *Bacilli bacterium* strain VT-13-104 isolated from the intestine of a patient with duodenal cancer. The genome is composed of 3,573,421 bp, with a G+C content of 35.7%. It possesses 3,254 predicted protein-coding genes encoding multidrug resistance transporters, resistance to antibiotics, and virulence factors.

## GENOME ANNOUNCEMENT

Bacteria belonging to the genus *Oceanobacillus* are motile, Gram positive, aerobic, and rod shaped. These bacteria have been isolated from deep-sea sediments, salt fields, and human feces (1–3). Sequencing of the complete 16S rRNA gene of *Bacilli bacterium* strain VT-13-104, which was isolated from the intestine of a patient with duodenal cancer, was found to possess 99% sequence identity with *Oceanobacillus caeni*, a bacterium first described in 2008 as an environmental microorganism found in wastewater (4). To date, *O. caeni* has not been found in humans.

Since biochemical and matrix-assisted laser desorption ionization–time of flight mass spectrometry (MALDI-TOF MS) analysis of *B. bacterium* strain VT-13-104 yielded divergent, low-discriminatory results, whole-genome sequencing of the bacterium was performed. The whole genome was determined using Illumina HiSeq 2500 sequencing technology (Illumina GA IIx, Illumina, CA). Library preparation, sequencing reactions, and runs were carried out according to the manufacturer’s instructions. A total of 5,487,668 high-quality 125-bp paired-end reads were produced, resulting in an approximate coverage of 384×. *De novo* assembly was performed using the SPAdes assembler (version 3.5.0), which generated 80 contigs (5).

The assembled 80 contigs totaled 3,573,421 bp (max length, 393,299), with a G+C content of 35.7%. Sequence annotation was performed using the NCBI Prokaryotic Genome Annotation Pipeline (6). There are 79 tRNA genes, 18 rRNA operons, 1 noncoding RNA (ncRNA) operon, and 3,254 protein coding sequences (CDSs) (934-bp average length) in the genome sequence. The analysis revealed multidrug resistance transporters of the ABC, MATE, MgtE, and MFS families, genes encoding resistance to antibiotics, including fosmidomycin, fosfomycin (*fosB*), quinolone, bacitracin (*bacA*), chloramphenicol, and the acriflavin resistance gene. The genome contains previously known virulence factors including adhesins and a RelE toxin in addition to capsular, flagellar, and sporulation proteins (7–9).

In comparison to the genome of *O. massiliensis*, the phylogenetically closest full genome sequence available, *B. bacterium* strain VT-13-104 is larger (3,573,421 bp versus 3,532,675 bp), has a lower G+C content (35.7% versus 40.35%), and fewer protein-coding genes (3,254 versus 3,519). An *in silico* DNA-DNA hybridization (DDH) analysis of the genomes of both *B. bacterium* VT-13-104 and *O. massiliensis* using the Genome-to-Genome Distance Calculator (GGDC 2.0) algorithm produced a DDH value of 42.10%, which is well below the threshold value of 70% set for genomes belonging to the same species (10, 11).

The complete genome sequence of *B. bacterium* VT-13-104 and its characterization will allow for a better understanding of its role in patients with duodenal cancer, as well as the host interaction and pathogenicity of this bacterium.

### Nucleotide sequence accession number.

The complete genome sequence has been deposited in the NCBI database under the accession no. LAZH00000000.
